# Bimodal age distribution at diagnosis in breast cancer persists across molecular and genomic classifications

**DOI:** 10.1007/s10549-019-05442-2

**Published:** 2019-09-18

**Authors:** Emma H. Allott, Yue Shan, Mengjie Chen, Xuezheng Sun, Susana Garcia-Recio, Erin L. Kirk, Andrew F. Olshan, Joseph Geradts, H. Shelton Earp, Lisa A. Carey, Charles M. Perou, Ruth M. Pfeiffer, William F. Anderson, Melissa A. Troester

**Affiliations:** 1 Centre for Cancer Research and Cell Biology, Queen’s University Belfast, Belfast, UK; 2 Department of Biostatistics, University of North Carolina at Chapel Hill, Chapel Hill, NC USA; 3 Departments of Medicine and Human Genetics, University of Chicago, Chicago, IL USA; 4 Department of Epidemiology, University of North Carolina at Chapel Hill, Chapel Hill, NC USA; 5 Lineberger Comprehensive Cancer Center, University of North Carolina at Chapel Hill, Chapel Hill, NC USA; 6 Department of Population Sciences, City of Hope, Duarte, CA USA; 7 Department of Medicine, University of North Carolina at Chapel Hill, Chapel Hill, NC USA; 8 Division of Cancer Epidemiology and Genetics, National Cancer Institute, Bethesda, MD USA; 9 Centre for Cancer Research and Cell Biology, Queen’s University Belfast, Health Sciences Building, Room 2.12, 97 Lisburn Road, Belfast, BT9 7AE Northern Ireland UK; 10 Department of Epidemiology, University of North Carolina at Chapel Hill, CB 7435, 135 Dauer Drive, Chapel Hill, NC 27599 USA

**Keywords:** Bimodality, Estrogen receptor, Etiology, Mixture model, Race, Subtype, PAM50

## Abstract

**Purpose:**

Female breast cancer demonstrates bimodal age frequency distribution patterns at diagnosis, interpretable as two main etiologic subtypes or groupings of tumors with shared risk factors. While RNA-based methods including PAM50 have identified well-established clinical subtypes, age distribution patterns at diagnosis as a proxy for etiologic subtype are not established for molecular and genomic tumor classifications.

**Methods:**

We evaluated smoothed age frequency distributions at diagnosis for Carolina Breast Cancer Study cases within immunohistochemistry-based and RNA-based expression categories. Akaike information criterion (AIC) values compared the fit of single density versus two-component mixture models. Two-component mixture models estimated the proportion of early-onset and late-onset categories by immunohistochemistry-based ER (*n* = 2860), and by RNA-based *ESR1* and PAM50 subtype (*n* = 1965). PAM50 findings were validated using pooled publicly available data (*n* = 8103).

**Results:**

Breast cancers were best characterized by bimodal age distribution at diagnosis with incidence peaks near 45 and 65 years, regardless of molecular characteristics. However, proportional composition of early-onset and late-onset age distributions varied by molecular and genomic characteristics. Higher ER-protein and *ESR1*-RNA categories showed a greater proportion of late age-at-onset. Similarly, PAM50 subtypes showed a shifting age-at-onset distribution, with most pronounced early-onset and late-onset peaks found in Basal-like and Luminal A, respectively.

**Conclusions:**

Bimodal age distribution at diagnosis was detected in the Carolina Breast Cancer Study, similar to national cancer registry data. Our data support two fundamental age-defined etiologic breast cancer subtypes that persist across molecular and genomic characteristics. Better criteria to distinguish etiologic subtypes could improve understanding of breast cancer etiology and contribute to prevention efforts.

**Electronic supplementary material:**

The online version of this article (10.1007/s10549-019-05442-2) contains supplementary material, which is available to authorized users.

## Introduction

Breast cancer heterogeneity may obscure etiologic risk factor associations if tumor subtypes are inadequately or incorrectly classified [1]. Etiologic studies generally group breast cancer into two or more protein-based subtypes using immunohistochemistry expression of estrogen receptor (ER), progesterone receptor (PR), and HER2 [2]. On the other hand, efforts to classify breast cancer into four genomic-intrinsic subtypes have focused on determining targeted therapies and cancer-specific clinical outcomes [3]. However, for cancer prevention efforts, optimizing subtype classification for etiologic subtypes is the key for understanding risk factor associations.

There is emerging evidence, based on bimodal age frequency distributions at diagnosis, that breast cancer can be divided into just two etiologically distinct subtypes [4]. Breast cancer bimodality has been observed across categories of ER status, tumor characteristics and histologic subtypes [5]. Bimodality has also been observed in different populations, for example, in both black and white breast cancer cases in the US [6] and South Africa [7]. However, prior evidence for breast cancer bimodality has been based on national cancer registries, which lack detailed molecular and genomic data. No studies, to our knowledge, have comprehensively explored evidence for bimodal age distribution at diagnosis across quantitative protein-based (i.e., percent ER-positivity) or RNA-based (i.e., *ESR1* and PAM50) tumor characteristics.

Using data from the Carolina Breast Cancer Study, we visualized age distributions at diagnosis and applied two-component mixture models across categories of breast cancer cases defined by molecular and genomic characteristics. We also sought to identify molecular or genomic features that could separate etiologically distinct breast cancers into single or unimodal age distributions at diagnosis.

## Methods

### Study design and participants

The Carolina Breast Cancer Study is a case–control study conducted in North Carolina (NC) in three phases (Phase 1: 1993–1996, Phase 2: 1996–2001 and Phase 3: 2008–2013), the details of which have been described previously [8]. Briefly, invasive breast cancer cases in women between 20 and 74 years of age were identified using rapid case ascertainment in cooperation with the NC Central Cancer Registry, and African American and young cases (aged 20–49 years) were oversampled [8]. The study was approved by the Office of Human Research Ethics at the University of North Carolina at Chapel Hill (UNC) and written informed consent was obtained from each participant. We used data from all invasive breast cancer cases across all three Carolina Breast Cancer Study phases (n = 4806) for the present analysis (Supplementary Table 1). Tumor size and lymph node status were abstracted from medical records, and these data were available for *n* = 4618 (96%) and *n* = 4751 (99%) study participants, respectively. Combined grade was centrally assigned by a single breast cancer pathologist (JG) using the Nottingham breast cancer grading system [9], and was available for *n* = 3408 (71%) cases.

### Immunohistochemistry analyses

All quantitative ER protein data, available for a total of *n* = 2860 (60%) cases (Supplementary Table 1), were obtained from immunohistochemistry staining. Immunohistochemistry expression of ER was abstracted from the medical records for *n* = 496 cases from Phases 1 and 2. For the remainder (*n* = 206 cases from Phases 1 and 2, and *n* = 2158 cases from Phase 3), formalin-fixed paraffin-embedded tumor blocks were requested from participating pathology laboratories. Tumor blocks were used to generate whole sections for all cases in Phases 1 and 2, and for 473 (22%) cases in Phase 3. For the remainder of cases in Phase 3 (*n* = 1685, 78%), tumor blocks were used to generate tissue microarrays, as previously described [2]. Immunohistochemistry staining was performed at the Immunohistochemistry Core Laboratory at UNC, and quantified using automated image analysis, as previously described [2]. When data were combined across Phases, demographic and tumor characteristics of cases with and without quantitative ER were similar (Supplementary Table 1).

Among ER-positive cases, we categorized ER expression as borderline (≥ 1–< 10%), low (≥ 10–< 40%), intermediate (≥ 40–< 80%), high (≥ 80%), and very high (≥ 95%). Expression categories were selected to be in line with a previous study [10] and to avoid sparse sample sizes in any given category.

### Genomic analyses

Nanostring assays were used to measure PAM50 gene signature [11], which includes *ESR1* gene, on *n* = 1965 cases from the Carolina Breast Cancer Study. Assays were performed in the Rapid Adoption Molecular (RAM) laboratory at UNC as previously described [12, 13]. Cases with and without RNA data had similar demographic and tumor characteristics (Supplementary Table 1). PAM50 subtype was determined using a similarity-to-centroid approach as previously described [11, 13] to classify breast tumors into four intrinsic subtypes (Luminal A, Luminal B, HER2-enriched, Basal-like). Tumors classified as normal-like (*n* = 66) were excluded from our analysis, given that this classification is thought to arise from extensive normal epithelial or stromal content in the tumor [14]. *ESR1* gene expression was median-centered and standardized to zero mean and unit variance, then categorized into quartiles based on expression levels in all cases.

We assembled a large validation genomics dataset of invasive breast cancer cases (*n* = 8103) by pooling publicly available data from The Cancer Genome Atlas (TCGA) [15], the Molecular Taxonomy of Breast Cancer International Consortium (METABRIC; EGAS00000000083) [16, 17], the Sweden Cancerome Analysis Network-Breast Initiative (SCAN-B; GSE81538 and GSE96058) [18], the UNC337 dataset from the UNC Microarray Database (GSE18229) [19], a previously pooled set of human breast tumors (GSE26338, GSE2034, GSE12276, GSE2603) [20] and the MD Anderson Cancer Center dataset (MDACC; GSE25066) [21]. PAM50 subtype was assigned in the validation cohort as described above for the Carolina Breast Cancer Study.

### Statistical analysis

We constructed smoothed age frequency distributions at diagnosis (i.e., density plots) across categories of ER protein expression, across quartiles of *ESR1* gene expression, and according to the intrinsic PAM50 subtype. Within each category defined by molecular or genomic characteristics, we assessed the performance of a single density model versus a two-component mixture model. We explored two different parameterizations of the data: a normal density, and a semi-nonparametric density with a polynomial component to allow for skewness and heavy tails in the distributions. Single density and two-component mixture models were each evaluated using normal density and semi-nonparametric density parameters, producing a total of four models for comparison within each molecular or genomic category. Models were compared using Akaike information criterion (AIC) values, with smaller AIC values indicating a better fit. Using this approach, we first identified the top-ranking single density model and the top-ranking two-component mixture model, and we then compared the goodness of fit between these two models using the difference in their AIC values (Δ_AIC_), with Δ_AIC_ > 10 indicating a substantial difference in the goodness of fit between the two models and a Δ_AIC_ 4–10 indicating a difference in the goodness of fit between the models, albeit with slightly less confidence than a value > 10 [22]. For all categories determined to be bimodal, two-component statistical mixture models were applied to estimate the mixing proportion of early-onset (*p*-*early*) and late-onset (*p*-*late*) modes or peaks within each category, as previously described [5, 23].

Analysis was performed in the validation cohort as described above for the Carolina Breast Cancer Study.

Statistical analysis was conducted in SAS version 9.4 (SAS Institute, Cary, NC).

## Results

### Age distributions at diagnosis by ER and ESR1 expression level

**Fig. 1:**
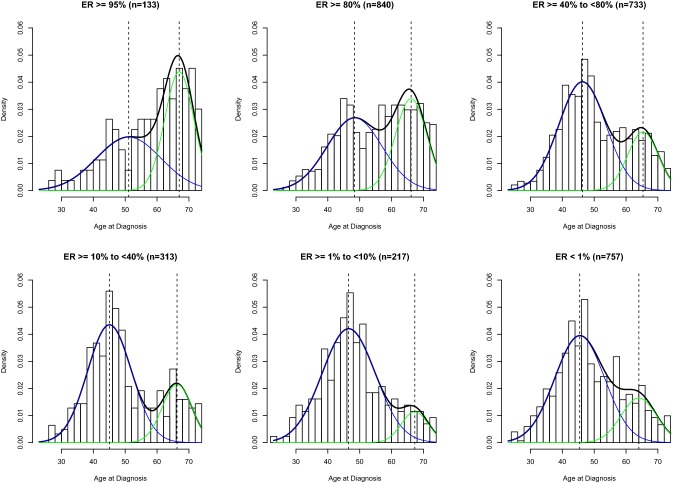
Density plots showing age frequency at diagnosis for invasive breast cancer cases from the Carolina Breast Cancer Study across immunohistochemistry-based ER categories

As illustrated in Fig. 1, the age distribution at breast cancer diagnosis showed a bimodal pattern in every ER category with early- and late-onset incidence peaks (or modes) near ages 45 and 65 years, respectively. While the proportion of cases within the late-onset peak decreased across decreasing categories of ER expression (Fig. 1, green line), the modal ages remained unchanged near 45 and 65 years. ER-negative cases (< 1% ER) and those with borderline (≥ 1–< 10%), low (≥ 10–< 40%), and intermediate (≥ 40–< 80%) ER expression levels had predominant early-onset peaks. In contrast, cases with high (≥ 80% and ≥ 95%) ER expression had predominant late-onset peaks (blue line). A bimodal pattern with shifting age-at-onset distributions but stable modes near 45 and 65 years was also observed when different clinical definitions of ER-positive status (i.e., ≥ 1% vs. ≥ 10%) were considered (Supplementary Fig. 1 and Supplementary Table 2).

Consistent with bimodal age distributions at diagnosis for all density plots (Fig. 1), a two-component mixture model fit the data better than a single density for all categories of ER expression (Table 1). As shown in Table 1, the majority of Δ_AIC_ values were greater than 10, indicating substantial confidence that the two-component mixture model provided the best fit for our data. One exception was noted for the ER-borderline group, where Δ_AIC_ lay between 4 and 10, still indicating that the two-component mixture model provided better fit, albeit with a slightly lower certainty. Cases with lower ER levels had a greater probability of belonging to the early-onset than the late-onset age distribution (e.g., *p*-*early *= 0.77, 0.88, 0.75, and 0.74 for cases with negative, borderline, low and intermediate ER expression, respectively). The proportion of early-onset age distribution further declined for cases with high and very high ER expression (e.g., *p*-*early *= 0.58 and *p*-*early *= 0.50, for cases with ≥ 80% and ≥ 95% ER expression levels, respectively). While ER expression level affected the mixing proportion, the early- and late-onset modes remained unchanged near ages 45 and 65.

**Table 1:** Comparison of single density versus two-component mixture model fit across molecular tumor categories in Carolina Breast Cancer Study cases, and estimates for early-onset and late-onset modes and mixing proportions for the selected model

	Total cases, n (%)	Median age at diagnosis (years)	Model fit (AIC)			Mode^b^ (years)		Mixing proportion^b^	
			AIC_single density_	AIC_two-component mixture_	Δ_AIC_^a^ (AIC_single_ − AIC_mixture_)	Early onset	Late onset	Early onset	Late onset
Protein-based categories									
Overall	2860	50	21,947.02	21,657.60	289.42	46	67	0.72	0.28
ER protein expression									
≥ 95%	133 (5)	62	1022.84	998.24	24.60	51	67	0.50	0.50
≥ 80%	840 (29)	57	6455.88	6362.56	93.32	48	66	0.58	0.42
≥ 40–< 80%	733 (26)	49	5560.16	5489.42	70.74	46	65	0.74	0.26
≥ 10–< 40%	313 (11)	48	2396.44	2343.82	52.62	45	66	0.75	0.25
≥ 1–< 10%	217 (8)	48	1637.76	1629.84	7.92	47	67	0.88	0.12
< 1%	757 (26)	48	5728.58	5695.14	33.44	45	64	0.77	0.23
RNA-based categories									
Overall	1965	49	15,092.82	14,899.46	193.36	47	67	0.76	0.24
ESR1 gene expression									
Quartile 4	492 (25)	53	3792.24	3722.54	69.70	48	67	0.69	0.31
Quartile 3	491 (25)	49	3738.00	3676.14	61.86	47	66	0.75	0.25
Quartile 2	491 (25)	49	3808.52	3770.84	37.68	47	67	0.78	0.22
Quartile 1	491 (25)	48	3730.72	3714.70	16.02	47	67	0.87	0.13
PAM50 subtype									
Luminal A	898 (47)	53	6886.04	6757.64	128.40	48	67	0.70	0.30
Luminal B	269 (14)	48	2071.28	2054.12	17.16	45	65	0.74	0.26
Her2-enriched	174 (9)	48	1328.06	1318.02	10.04	47	67	0.84	0.16
Basal-like	558 (29)	47	4260.84	4239.44	21.40	47	69	0.89	0.11

^a^Positive values favor the two-component mixture model and negative values favor the single density model, with Δ_AIC_ 4–10 indicating little support for the lower-ranking model and Δ_AIC_ > 10 indicating essentially no support for the lower-ranking model [22]

^b^Modes and mixing proportions are shown for the two-component mixture model, found to provide the best fit for all categories shown

Similar patterns were observed in race-stratified analyses (Supplementary Figs. 2, 3 and Supplementary Table 2), albeit with more pronounced early-onset peaks in black vs. white women both overall (e.g., *p*-*early *= 0.66 for white women, *p*-*early *= 0.75 for black women) and by ER status (e.g., *p*-*early *= 0.61 and 0.79 for cases with ER ≥ 10% and < 10% in white women, *p*-*early *= 0.72 and 0.83 for cases with ER ≥ 10% and < 10% in black women).

**Fig. 2:**
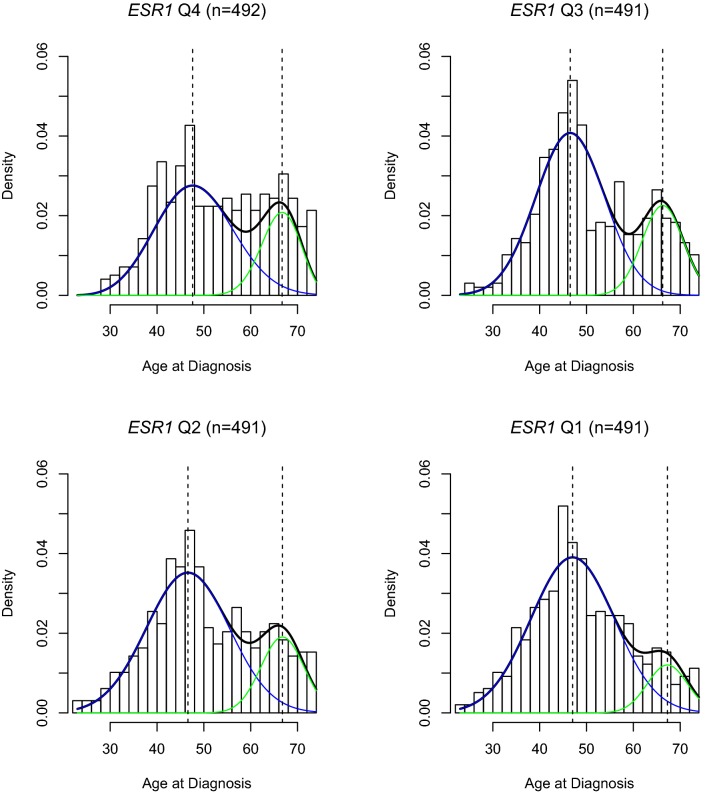
Density plots showing age frequency at diagnosis for invasive breast cancer cases from the Carolina Breast Cancer Study across RNA-based *ESR1* quartiles

As shown in Fig. 2, classifying cases by quartiles of *ESR1* gene expression recapitulated bimodal age distributions at diagnosis observed across categories of immunohistochemistry-based ER expression levels. While modal ages remained constant near ages 45 and 65, the proportion of late-onset cases gradually decreased across decreasing quartiles of *ESR1* expression (Q4 *p*-*late *= 0.31, Q3 *p*-*late *= 0.25, Q2 *p*-*late *= 0.22, Q1 *p*-*late *= 0.13; as shown in Table 1).

### Age distributions at diagnosis by genomic and tumor characteristics

**Fig. 3:**
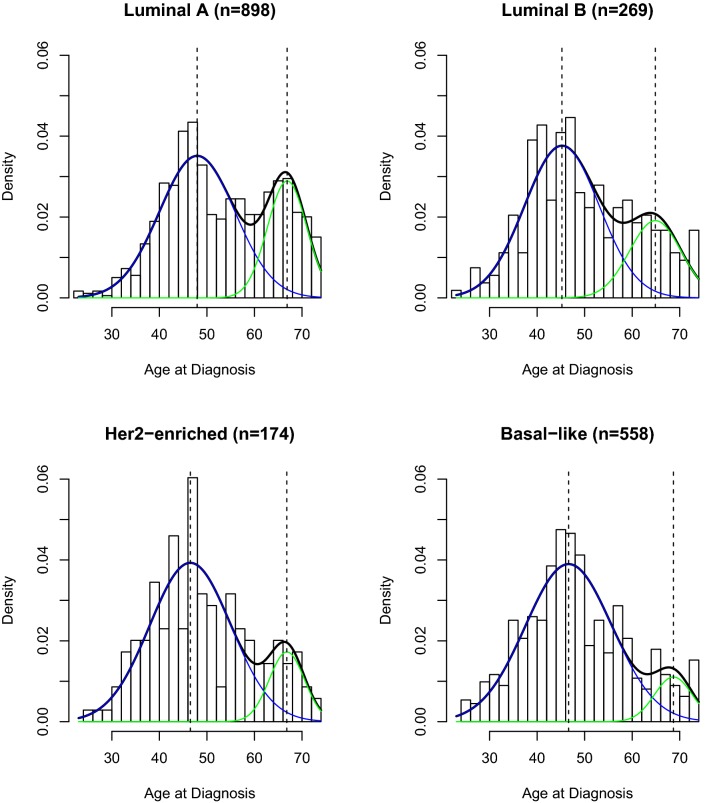
Density plots showing age frequency at diagnosis for invasive breast cancer cases from the Carolina Breast Cancer Study across PAM50 intrinsic subtype

In Fig. 3, we show that age at diagnosis was bimodally distributed for all PAM50 intrinsic subtypes. Basal-like, HER2-enriched, and Luminal B cancers all had predominant early-onset peaks with minor late-onset peaks, whereas the late-onset peak was most pronounced in Luminal A cancers.

In keeping with the bimodal appearance of the density plots, we found that each intrinsic subtype was best described by a two-component mixture model (Table 1). The proportion of late-onset age distribution decreased across intrinsic subtype categories, from Luminal A (*p*-*late *= 0.30), Luminal B (*p*-*late *= 0.26), HER2-enriched (*p*-*late *= 0.16), with Basal-like showing the lowest probability of late-onset disease (*p*-*late *= 0.11; Table 1). As we observed for ER and *ESR1* expression, while the mixing proportion was affected by intrinsic tumor subtype, the early- and late-onset modal ages remained near ages 45 and 65. As shown in Table 1, the majority of Δ_AIC_ values were greater than 10, indicating substantial confidence that the two-component mixture model provided the best fit for our data. One exception was noted for the HER2-enriched subtype, where Δ_AIC_ was equal to 10, still indicating that the two-component mixture model provided better fit, albeit with a slightly lower certainty. Similar patterns were observed in a large validation dataset of > 8000 invasive breast cancers, where all PAM50 subtypes were bimodally distributed with a declining probability of late-onset from Luminal A to Luminal B to HER2-enriched to Basal-like, mirrored by an increasing probability of early-onset across these subtypes (Supplementary Fig. 4 and Supplementary Table 3).

Likewise, all tumors categorized by high-risk and low-risk tumor characteristics were best described by a two-component mixture model, with modal ages near 45 and 65 years (Supplementary Fig. 5). The proportion of early-onset cancers increased with increasing combined grade. Similarly, larger tumors and tumors with multiple positive lymph nodes were more likely to belong to the early-onset distribution, compared to smaller and node-negative tumors (Supplementary Table 4).

Combined categories of molecular, genomic and tumor characteristics also failed to isolate a single population, with every combination best described by a two-component mixture model (data not shown).

## Discussion

The identification of at least four distinct intrinsic breast cancer subtypes [24] has guided the development of targeted therapy and contributed to improved breast cancer survival rates. However, it has been posited that breast cancer is comprised of just two etiologically distinct groups [4, 25], with ER status currently serving as the most widely used surrogate of these two subtypes [26]. Though not optimized for this purpose, classifying tumors by ER status has advanced our understanding of breast cancer risk factors. For example, increasing parity is inversely associated with risk of ER-positive breast cancer but positively associated with risk of ER-negative breast cancer, an effect that can be partially offset by breastfeeding [27, 28]. Under this proposed model, breast cancer is a two-component mixture of ER-positive and ER-negative tumor populations [4], with differences in quantitative levels of ER expression reflecting enrichment for one or other population. Building on this hypothesis, our work in the Carolina Breast Cancer Study shows that breast cancer bimodality is a robust characteristic observed across molecular and genomic tumor features.

Prior research using publicly available registry data from the US [5], as well as data from Europe [29], Africa [7, 30], and Asia [31], has established bimodal age at diagnosis as a universal feature of female breast cancer. Breast cancer bimodality has also been observed within categories defined by ER status [4], high-risk and low-risk tumor characteristics [32] and histologic subtype [5]. A notable exception to this bimodal age distribution at diagnosis is medullary carcinoma [5], a rare early-onset sporadic breast cancer that is linked to ER-negative and triple negative cancers, Basal-like tumor features [33], and the *BRCA1* mutation [34]. While developments in molecular and genomic tumor profiling technologies have advanced the field of breast cancer subtyping for prognosis and prediction, national cancer registries are limited to tumor characteristics reported in medical records and therefore lack these data. In the present study, we used quantitative ER expression and RNA data from the Carolina Breast Cancer Study to explore evidence for bimodality within groups defined by molecular and genomic features. We report that although certain molecular and genomic tumor characteristics enriched for either early-onset or late-onset breast cancer, we were unable to separate early-onset from late-onset breast cancer using existing molecular or genomic classifications, or any combinations thereof.

Interpretation of quantitative immunohistochemistry-based ER levels has been subject to some controversy. Replacement of the radio ligand-binding assay with immunohistochemistry for measuring ER status was accompanied by observations that ER expression was bimodally distributed [35]. Rimm and others have argued that the bimodal distribution of ER expression is an artifact of immunohistochemistry staining methods, which have been optimized to maximize the sensitivity of the assay [36, 37]. Indeed, we have observed a greater dynamic range of *ESR1* expression, compared to that of immunohistochemistry-based ER expression which becomes saturated at higher levels of *ESR1* [38]. However, several studies have since shown evidence for bimodal distribution not only of quantitative immunohistochemistry-based ER expression [39] but also of *ESR1* levels [39, 40], which are not subject to concerns regarding immunohistochemistry methodology. Herein, we build on evidence for breast cancer bimodality by showing bimodal age-at-incidence across categories of immunohistochemistry-based ER expression, *ESR1* levels, as well as PAM50 intrinsic subtype. As such, this manuscript bolsters evidence for bimodal age distribution at diagnosis as a universal characteristic of female breast cancer.

Breast cancer bimodality is consistent with tumors being derived from two distinct progenitor cell types, basal/myoepithelial versus luminal [4]. Large-scale genomic analyses have recently challenged the classification of cancers according to their tissue-of-origin. Using multi-platform genomic analyses, Hoadley et al. found that although most tumor types were classified by tissue-of-origin, several distinct cancer types converged into common subtypes regardless of tissue-of-origin, while others diverged into multiple subtypes within the tissue-of-origin classifications [25]. Breast cancer provided one of the most striking examples of this divergence, with Luminal/HER2-enriched and Basal-like breast cancers forming separate clusters as distinct from each other as from other tissue-of-origin cancer types (e.g., lung). Moreover, this integrated analysis revealed that marked molecular differences were observed between epithelial tumors arising from basal cell versus secretory cell types, suggesting that cell type-of-origin dominates molecular taxonomy of breast and other cancer types [25]. Shared etiology across cancers with different tissue-of-origin but shared cell type-of-origin (e.g., smoking as a shared risk factor for squamous bladder, head and neck and non-small cell lung cancers) may highlight the importance of classifying breast cancer according to the cell type-of-origin for understanding breast cancer etiology. Future studies should pursue the identification of molecular characteristics that can separate etiologically distinct subtypes of breast cancer.

Our findings should be considered in the context of several limitations. First, though a population-based study, the Carolina Breast Cancer Study oversampled for young and African American women. Our analysis did not account for this sampling schema and thus our population distribution is shifted toward younger ages relative to national distributions of breast cancer incidence. This is highlighted by our finding that modal ages for early and late-onset distributions lie at ages 45 and 65, whereas data from SEER breast cancer cases show that modes are closer to 50 and 70 years of age [5]. Restricting SEER data to the age range of the Carolina Breast Cancer Study produced similar bimodal age distributions at diagnosis (data not shown), suggesting that the slightly younger modes in the Carolina Breast Cancer Study may be due to the restricted age range at breast cancer diagnosis in the Carolina Breast Cancer Study (20–74) versus SEER (currently 10–117). However, rather than the absolute modal age which depends on the age distribution in the underlying population, the key attribute of these modes is that they are stable across molecular categories. Second, lower numbers of cases particularly in ER-borderline (ER 1–10%) and HER2-enriched categories may have limited our ability to discriminate between single density and two-component mixture models, as evidenced by Δ_AIC_ values between 4 and 10. However, Δ_AIC_ values in this range still provide support for a bimodal age distribution at diagnosis for these subgroups, albeit with a slightly lower certainty than when Δ_AIC_ is greater than 10 [22]. Third, although we had insufficient sample size to perform race-stratified analysis for each of the molecular subtypes, we were able to perform race-stratified analyses both overall and by ER status. Indeed, our race-stratified results are in agreement with findings from SEER analyses [5, 6], showing a larger proportion of early-onset cases among black women. Strengths of this study include the large number of African American women, a racial group disproportionately affected by high-risk breast cancer [12], as well as access to high quality molecular and genomic data for a large number of breast cancer cases.

## Conclusions

Using data from the Carolina Breast Cancer Study, we found evidence for a bimodal age distribution at breast cancer diagnosis both overall and within categories defined by molecular and genomic characteristics. While tumor subgroups defined by high-risk features (e.g., low immunohistochemistry-based ER levels, low *ESR1* expression, Basal-like subtype) showed enrichment for early-onset breast cancer, all of these categories and combinations thereof were best described by a two-component mixture model. Better criteria to distinguish these two etiologic subtypes could advance our understanding of breast cancer risk factors and inform prevention efforts.

## Electronic supplementary material

Below is the link to the electronic supplementary material.

**Supplementary Table** **1:** Demographic and tumor characteristics of Carolina Breast Cancer Study cases, overall and restricted to those with protein and RNA data. Supplementary material 1 (DOCX 13 kb)

**Supplementary Table** **2:** Comparison of single density versus two-component mixture model fit by ER status of Carolina Breast Cancer Study cases overall and by race, and estimates for early-onset and late-onset modes and mixing proportions for the selected model. Supplementary material 2 (DOCX 16 kb)

**Supplementary Table** **3:** Estimates for early-onset and late-onset modes and mixing proportions, given PAM50 subtype, in a pooled publicly-available dataset of 8103 tumors. Supplementary material 3 (DOCX 13 kb)

**Supplementary Table** **4:** Comparison of single density versus two-component mixture model fit across tumor characteristics of Carolina Breast Cancer Study cases, and estimates for early-onset and late-onset modes and mixing proportions for the selected model. Supplementary material 4 (DOCX 14 kb)

Supplementary Fig. 1: Density plots showing age frequency at diagnosis for invasive breast cancer cases from the Carolina Breast Cancer Study overall and across clinical ER cut points. Supplementary material 5 (PDF 39 kb)

**Supplementary Fig.** **2:** Density plots showing age frequency at diagnosis for invasive breast cancer cases from the Carolina Breast Cancer Study overall and across clinical ER cut points among white women. Supplementary material 6 (PDF 37 kb)

**Supplementary Fig.** **3:** Density plots showing age frequency at diagnosis for invasive breast cancer cases from the Carolina Breast Cancer Study overall and across clinical ER cut points among black women. Supplementary material 7 (PDF 38 kb)

**Supplementary Fig.** **4:** Density plots showing age frequency at diagnosis for invasive breast cancer cases from a pooled publicly-available dataset of 8103 tumors, across PAM50 intrinsic subtype. Supplementary material 8 (PDF 55 kb)

**Supplementary Fig.** **5:** Density plots showing age frequency at diagnosis for invasive breast cancer cases from the Carolina Breast Cancer Study across tumor characteristics. Supplementary material 9 (PDF 67 kb)

## Data Availability

The datasets used and/or analyzed during the current study are available from the corresponding author on reasonable request.
