# Supplementary material for: Bimodal age distribution at diagnosis in breast cancer persists across molecular and genomic classifications
Source: Breast Cancer Res Treat. 2019 Sep 18;179(1):185–95. doi: 10.1007/s10549-019-05442-2 (PMC6985047; doi:10.1007/s10549-019-05442-2)
Supplement: Supplementary file 1 — Supplementary Table 1: Demographic and tumor characteristics of Carolina Breast Cancer Study cases, overall and restricted to those with protein and RNA data. Supplementary material 1 (DOCX 13 kb) [file 10549_2019_5442_MOESM1_ESM.docx]

**Supplementary Table 1:** Demographic and tumor characteristics of Carolina Breast Cancer Study cases, overall and restricted to those with protein and RNA data

|  | **All CBCS cases**  **(n=4,806)** | **CBCS cases with protein data (n=2,860)** | **CBCS cases with RNA data (n=1,965)** |
| --- | --- | --- | --- |
| **Age at diagnosis, mean (SD)** | 51.5 (11.3) | 51.9 (11.3) | 51.5 (11.3) |
| **Study Phase** |  |  |  |
| Phase 1 | 861 (18) | 259 (9) | 105 (5) |
| Phase 2 | 947 (20) | 443 (16) | 410 (21) |
| Phase 3 | 2,998 (62) | 2,158 (75) | 1,450 (74) |
| **Menopausal status, n (%)** |  |  |  |
| Pre | 2,217 (46) | 1,274 (45) | 919 (47) |
| Post | 2,589 (54) | 1,586 (55) | 1,046 (53) |
| **Race, n (%)** |  |  |  |
| White | 2,416 (50) | 1,439 (50) | 1,008 (51) |
| African American | 2,283 (48) | 1,350 (47) | 908 (46) |
| Other | 107 (2) | 71 (3) | 49 (3) |
| **Combined tumor grade** |  |  |  |
| I | 718 (21) | 491 (22) | 253 (17) |
| II | 1,243 (36) | 840 (37) | 536 (36) |
| III | 1,447 (42) | 926 (41) | 698 (47) |
| missing | 1,398 | 603 | 478 |
| **Tumor size (cm)** |  |  |  |
| ≤2 | 2,422 (52) | 1,547 (55) | 899 (47) |
| >2-5 | 1,672 (36) | 1,013 (36) | 800 (42) |
| >5 | 524 (11) | 258 (9) | 225 (12) |
| missing | 188 | 42 | 41 |
| **Lymph node status** |  |  |  |
| Negative | 2,901 (61) | 1,769 (62) | 1,134 (58) |
| Positive | 1,850^a^ (39) | 1,080 (38) | 826 (58) |
| missing | 55 | 11 | 5 |
| **Clinical ER status** |  |  |  |
| Negative | 1,623 (35) | 892 (31) | 681 (35) |
| Positive | 3,003 (65) | 1,965 (69) | 1,262 (65) |
| missing | 180 | 3 | 22 |
| **Clinical PR status** |  |  |  |
| Negative | 2,098 (46) | 1,233 (43) | 902 (47) |
| Positive | 2,508 (54) | 1,612 (57) | 1,032 (53) |
| missing | 200 | 15 | 31 |
| **Clinical HER2 status** |  |  |  |
| Negative | 3,620 (83) | 2,305 (84) | 1,628 (84) |
| Equivocal | 48 (1) | 34 (1) | 22 (1) |
| Positive | 686 (16) | 392 (14) | 288 (15) |
| missing | 452 | 129 | 27 |

^a^n=123 cases with positive lymph node status were missing information regarding number of positive lymph nodes and were therefore excluded from the relevant analysis
