# Supplementary material for: Bimodal age distribution at diagnosis in breast cancer persists across molecular and genomic classifications
Source: Breast Cancer Res Treat. 2019 Sep 18;179(1):185–95. doi: 10.1007/s10549-019-05442-2 (PMC6985047; doi:10.1007/s10549-019-05442-2)
Supplement: Supplementary file 2 — Supplementary Table 2: Comparison of single density versus two-component mixture model fit by ER status of Carolina Breast Cancer Study cases overall and by race, and estimates for early-onset and late-onset modes and mixing proportions for the selected model. Supplementary material 2 (DOCX 16 kb) [file 10549_2019_5442_MOESM2_ESM.docx]

**Supplementary Table 2:** Comparison of single density versus two-component mixture model fit by ER status of Carolina Breast Cancer Study cases overall and by race, and estimates for early-onset and late-onset modes and mixing proportions for the selected model

|  | **Total cases,**  **n (%)** | **Median age at diagnosis (years)** | **Model fit (AIC)** | | | | **Mode^b^ (years)** | | **Mixing proportion^b^** | |
| --- | --- | --- | --- | --- | --- | --- | --- | --- | --- | --- |
|  |  |  | **AIC_single density_** | **AIC_two-component mixture_** | **Δ_AIC_^a^**  **(AIC_single_ - AIC_mixture_)** | | **Early onset** | **Late onset** | **Early onset** | **Late onset** |
| **All women** |  |  |  |  |  | |  |  |  |  |
| **Overall** | 2,860 | 50 | 21947.02 | 21657.60 | 289.42 | | 46 | 67 | 0.72 | 0.28 |
| **ER status** |  |  |  |  |  | |  |  |  |  |
| ≥1% | 2,103 (74) | 51 | 16179.32 | 15925.88 | 253.44 | | 47 | 67 | 0.68 | 0.32 |
| <1% | 757 (26) | 48 | 5728.58 | 5695.14 | 33.44 | | 45 | 64 | 0.77 | 0.23 |
|  |  |  |  |  |  | |  |  |  |  |
| ≥10% | 1,886 (66) | 52 | 14520.18 | 14275.54 | 244.64 | | 46 | 67 | 0.65 | 0.35 |
| <10% | 974 (34) | 48 | 7360.96 | 7317.94 | 43.02 | | 46 | 65 | 0.82 | 0.18 |
|  |  |  |  |  |  | |  |  |  |  |
| **White women** |  |  |  |  | |  |  |  |  |  |

| **Overall** | 1,510 | 49 | 11605.6 | 11377.58 | 228.02 | 45 | 66 | 0.66 | 0.34 |
| --- | --- | --- | --- | --- | --- | --- | --- | --- | --- |

| **ER status** |  |  |  |  |  |  |  |  |  |
| --- | --- | --- | --- | --- | --- | --- | --- | --- | --- |
| ≥1% | 1,222 | 51 | 9391.88 | 9199.40 | 192.48 | 46 | 66 | 0.64 | 0.36 |
| <1% | 288 | 48 | 2195.90 | 2169.14 | 26.76 | 45 | 66 | 0.79 | 0.21 |
| ≥10% | 1,104 | 52 | 8500.78 | 8317.80 | 192.96 | 46 | 66 | 0.61 | 0.39 |
| <10% | 406 | 48 | 3071.00 | 3035.08 | 35.92 | 45 | 65 | 0.79 | 0.21 |
| **Black women** |  |  |  |  |  |  |  |  |  |

| **Overall** | 1,350 | 50 | 10339.98 | 10263.08 | 76.90 | 45 | 66 | 0.75 | 0.25 |
| --- | --- | --- | --- | --- | --- | --- | --- | --- | --- |

| **ER status** |  |  |  |  |  |  |  |  |  |
| --- | --- | --- | --- | --- | --- | --- | --- | --- | --- |
| ≥1% | 881 | 52 | 6788.78 | 6724.74 | 64.04 | 48 | 66 | 0.76 | 0.24 |
| <1% | 469 | 48 | 3538.04 | 3527.24 | 10.80 | 45 | 62 | 0.75 | 0.25 |
| ≥10% | 782 | 52 | 6016.12 | 5959.04 | 57.08 | 47 | 67 | 0.72 | 0.28 |
| <10% | 568 | 48 | 4295.70 | 4284.54 | 11.16 | 46 | 64 | 0.83 | 0.17 |

**^a^**positive values favor the two-component mixture model and negative values favor the single density model, with Δ_AIC_ >10 indicating essentially no support for the lower-ranking model^22^

^b^modes and mixing proportions are shown for the two-component mixture model, found to provide the best fit for all categories
