# Supplementary material for: Bimodal age distribution at diagnosis in breast cancer persists across molecular and genomic classifications
Source: Breast Cancer Res Treat. 2019 Sep 18;179(1):185–95. doi: 10.1007/s10549-019-05442-2 (PMC6985047; doi:10.1007/s10549-019-05442-2)
Supplement: Supplementary file 3 — Supplementary Table 3: Estimates for early-onset and late-onset modes and mixing proportions, given PAM50 subtype, in a pooled publicly-available dataset of 8103 tumors. Supplementary material 3 (DOCX 13 kb) [file 10549_2019_5442_MOESM3_ESM.docx]

**Supplementary Table 3:** Estimates for early-onset and late-onset modes and mixing proportions, given PAM50 subtype, in a pooled publicly-available dataset of 8,103 tumors

|  | **Total cases,**  **n (%)** | **Median age at diagnosis (years)** | **Model fit (AIC)** | | | **Mode^b^ (years)** | | **Mixing proportion^b^** | |
| --- | --- | --- | --- | --- | --- | --- | --- | --- | --- |
|  |  |  | **AIC_single density_** | **AIC_two-component mixture_** | **Δ_AIC_^a^ (AIC_single_ - AIC_mixture_)** | **Early onset** | **Late onset** | **Early onset** | **Late onset** |
| **Pooled dataset PAM50 subtype** |  |  |  |  |  |  |  |  |  |
| Overall | 8,103 | 60 | 65321.60 | 65041.16 | 280.44 | 46 | 65 | 0.26 | 0.74 |
| Luminal A | 3,292 | 62 | 26321.74 | 26188.12 | 133.62 | 47 | 66 | 0.32 | 0.68 |
| Luminal B | 1,750 | 63 | 14066.26 | 14008.40 | 69.02 | 46 | 68 | 0.29 | 0.71 |
| Her2-enriched | 917 | 58 | 7417.20 | 7399.70 | 17.50 | 49 | 69 | 0.54 | 0.46 |
| Basal-like | 1,411 | 53 | 11333.00 | 11312.34 | 20.66 | 44 | 61 | 0.42 | 0.58 |

**^a^**positive values favor the two-component mixture model and negative values favor the single density model, with Δ_AIC_ >10 indicating essentially no support for the lower-ranking model^22^

**^b^**modes and mixing proportions are shown for the two-component mixture model, found to provide the best fit for all categories
