# Supplementary material for: Bimodal age distribution at diagnosis in breast cancer persists across molecular and genomic classifications
Source: Breast Cancer Res Treat. 2019 Sep 18;179(1):185–95. doi: 10.1007/s10549-019-05442-2 (PMC6985047; doi:10.1007/s10549-019-05442-2)
Supplement: Supplementary file 4 — Supplementary Table 4: Comparison of single density versus two-component mixture model fit across tumor characteristics of Carolina Breast Cancer Study cases, and estimates for early-onset and late-onset modes and mixing proportions for the selected model. Supplementary material 4 (DOCX 14 kb) [file 10549_2019_5442_MOESM4_ESM.docx]

**Supplementary Table 4:** Comparison of single density versus two-component mixture model fit across tumor characteristics of Carolina Breast Cancer Study cases, and estimates for early-onset and late-onset modes and mixing proportions for the selected model

|  | **Total cases,**  **n (%)** | **Median age at diagnosis (years)** | **Model fit (AIC)** | | | **Mode^b^ (years)** | | **Mixing proportion^b^** | |
| --- | --- | --- | --- | --- | --- | --- | --- | --- | --- |
|  |  |  | **AIC_single density_** | **AIC_two-component mixture_** | **Δ_AIC_^a^ (AIC_single_ - AIC_mixture_)** | **Early onset** | **Late onset** | **Early onset** | **Late onset** |
| **Combined grade** |  |  |  |  |  |  |  |  |  |
| 1 | 718 (21) | 54 | 5481.84 | 5372.52 | 109.32 | 47 | 65 | 0.60 | 0.40 |
| 2 | 1,243 (36) | 51 | 9557.12 | 9420.14 | 136.98 | 46 | 65 | 0.65 | 0.35 |
| 3 | 1,447 (42) | 47 | 10982.76 | 10894.24 | 88.52 | 45 | 65 | 0.82 | 0.18 |
| **Tumor size** |  |  |  |  |  |  |  |  |  |
| ≤2 cm | 2,422 (52) | 52 | 18601.18 | 18280.02 | 321.16 | 47 | 65 | 0.66 | 0.34 |
| >2-<5 cm | 1,672 (36) | 48 | 12780.44 | 12655.96 | 124.48 | 44 | 66 | 0.76 | 0.24 |
| >5 cm | 524 (11) | 47 | 3983.08 | 3963.84 | 25.98 | 45 | 65 | 0.83 | 0.17 |
| **Lymph node status** |  |  |  |  |  |  |  |  |  |
| Negative | 2,901 (63) | 51 | 22336.6 | 21979.92 | 356.68 | 47 | 66 | 0.70 | 0.30 |
| 1-2 positive nodes | 901 (19) | 48 | 6885.48 | 6817.40 | 68.80 | 44 | 64 | 0.70 | 0.30 |
| >2 positive nodes | 826 (18) | 48 | 6289.22 | 6238.58 | 67.14 | 46 | 65 | 0.80 | 0.20 |

**^a^**values represent the largest AIC for single density vs. two-component mixture models, respectively

^b^positive values favor the two-component mixture model and negative values favor the single density model, with Δ_AIC_ >10 indicating essentially no support for the lower-ranking model^22^

**^c^**modes and mixing proportions are shown for the two-component mixture model, found to provide the best fit for all categories shown
