# Supplementary figures and images for: Bimodal age distribution at diagnosis in breast cancer persists across molecular and genomic classifications
Source: Breast Cancer Res Treat. 2019 Sep 18;179(1):185–95. doi: 10.1007/s10549-019-05442-2 (PMC6985047; doi:10.1007/s10549-019-05442-2)

All cases with ER (n=2860)

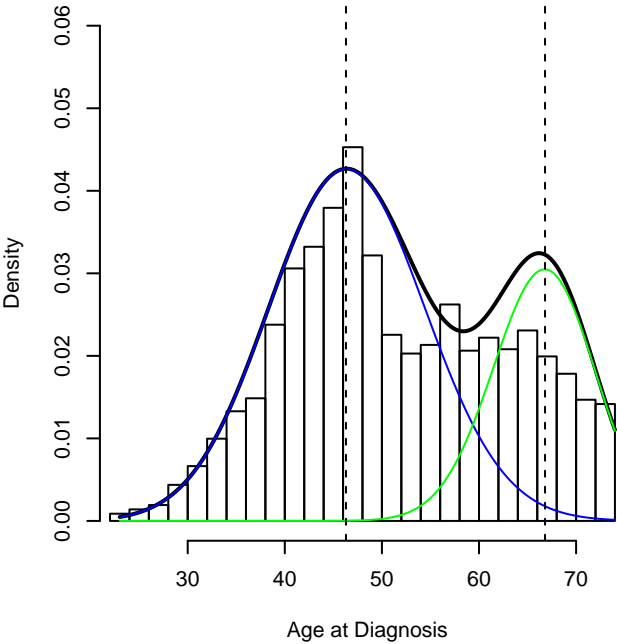

ER < 1% (n=757)

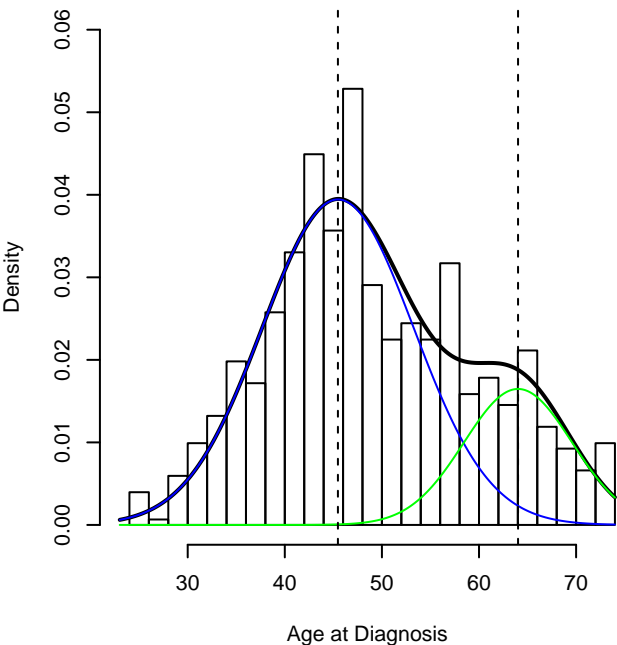

ER >= 1% (n=2103)

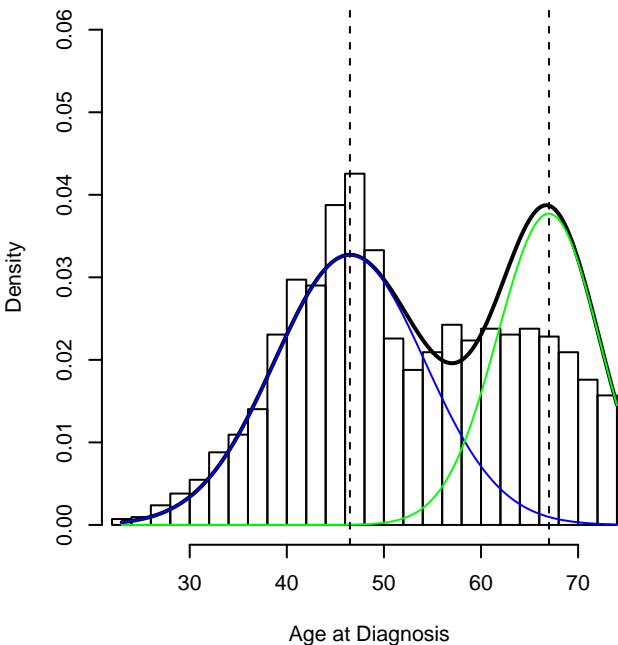

ER <10% (n=974)

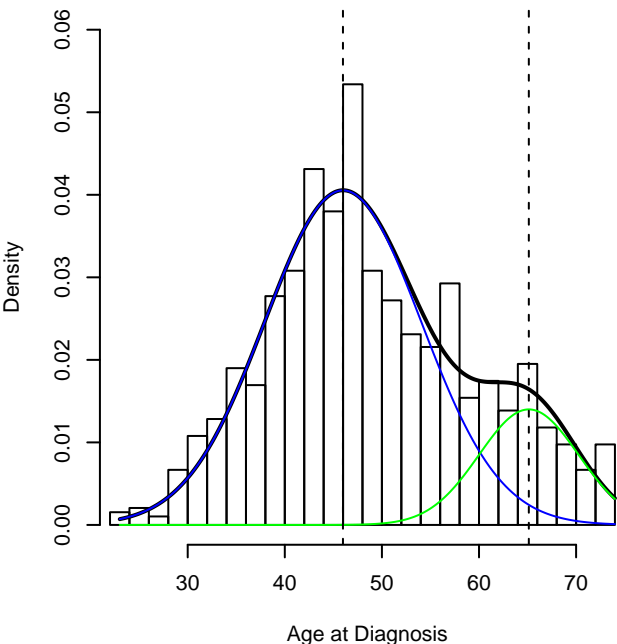

ER >= 10% (n=1886)

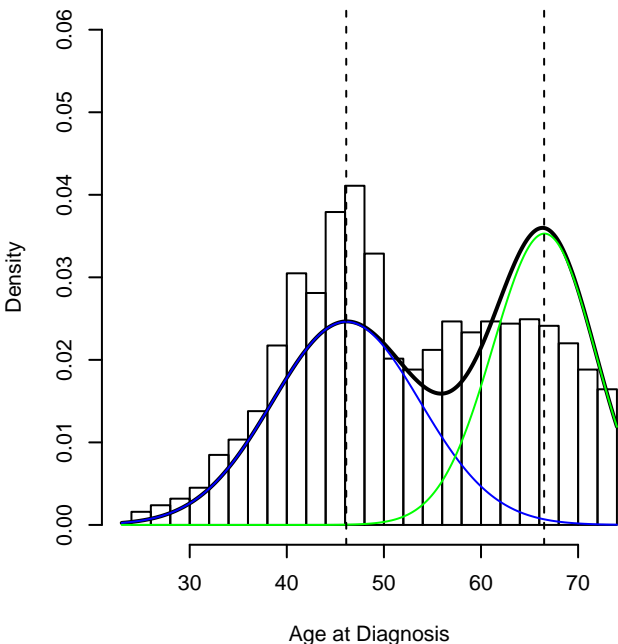

Supplement: Supplementary file 5 — Supplementary Fig. 1: Density plots showing age frequency at diagnosis for invasive breast cancer cases from the Carolina Breast Cancer Study overall and across clinical ER cut points. Supplementary material 5 (PDF 39 kb) [file 10549_2019_5442_MOESM5_ESM.pdf]

All cases with ER (n=1510)

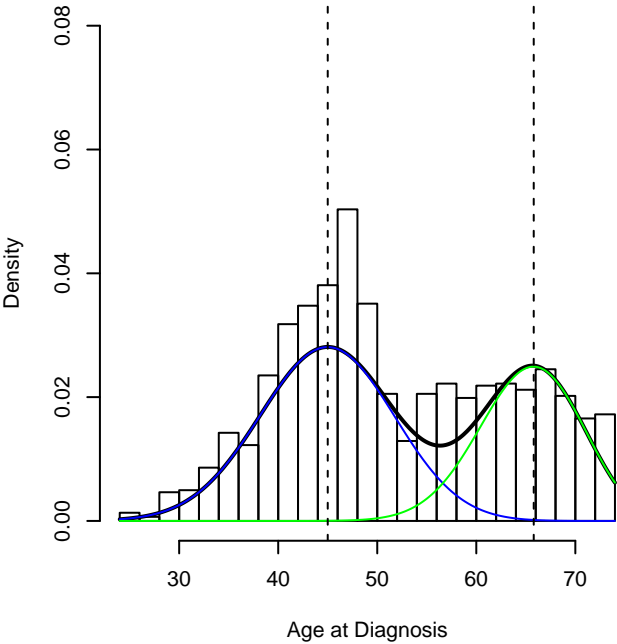

ER < 1% (n=288)

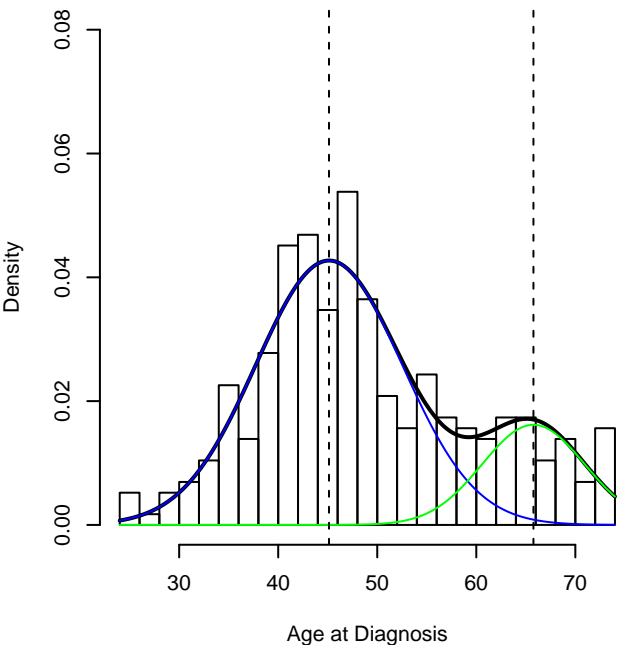

ER >= 1% (n=1222)

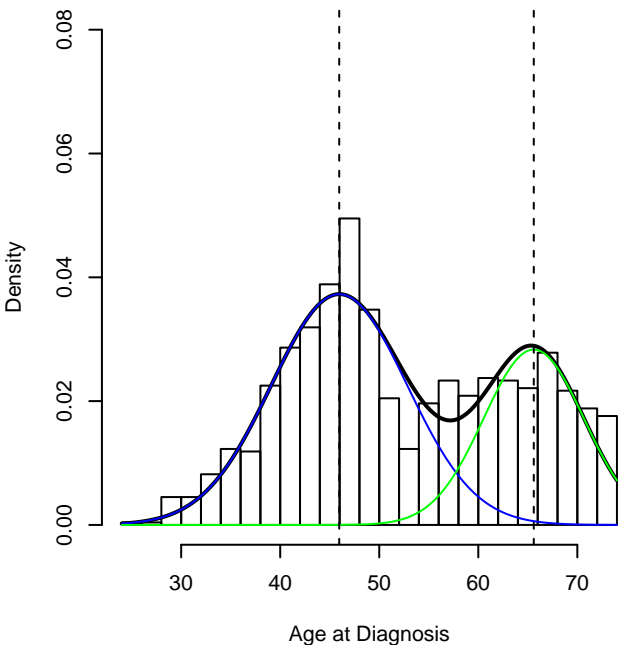

ER <10% (n=406)

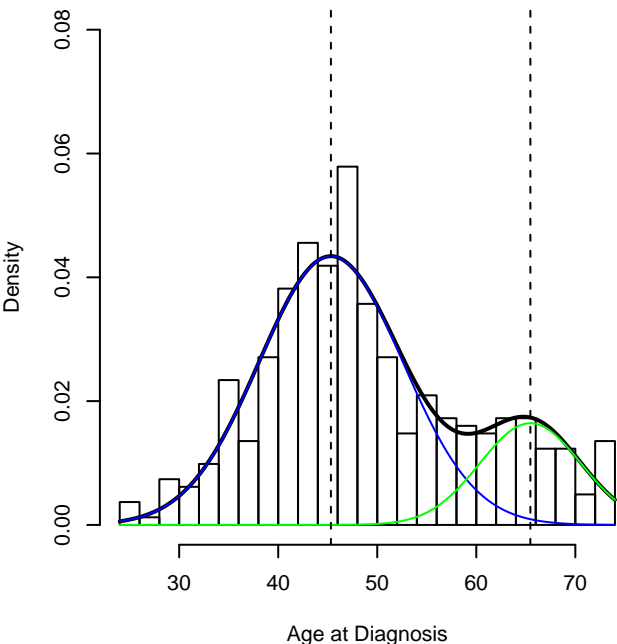

ER >= 10% (n=1104)

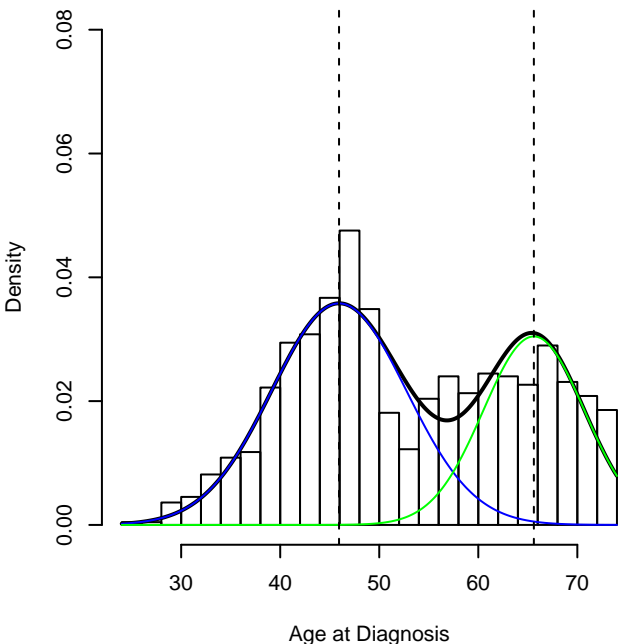

Supplement: Supplementary file 6 — Supplementary Fig. 2: Density plots showing age frequency at diagnosis for invasive breast cancer cases from the Carolina Breast Cancer Study overall and across clinical ER cut points among white women. Supplementary material 6 (PDF 37 kb) [file 10549_2019_5442_MOESM6_ESM.pdf]

All cases with ER (n=1350)

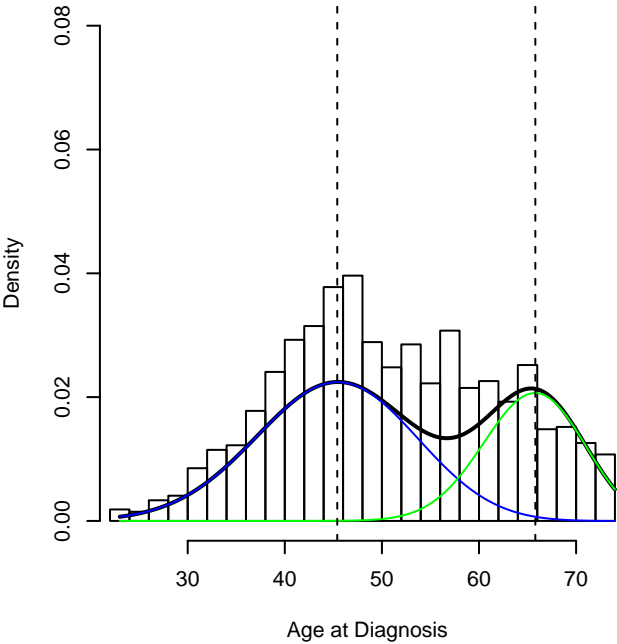

ER < 1% (n=469)

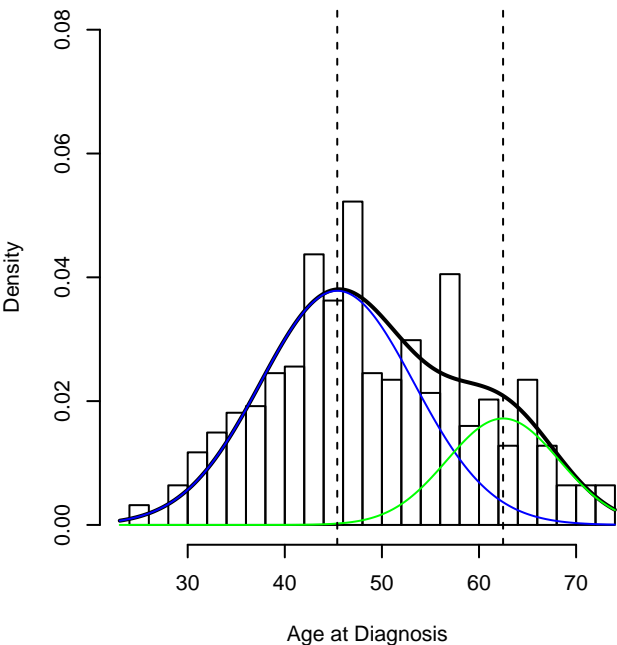

ER >= 1% (n=881)

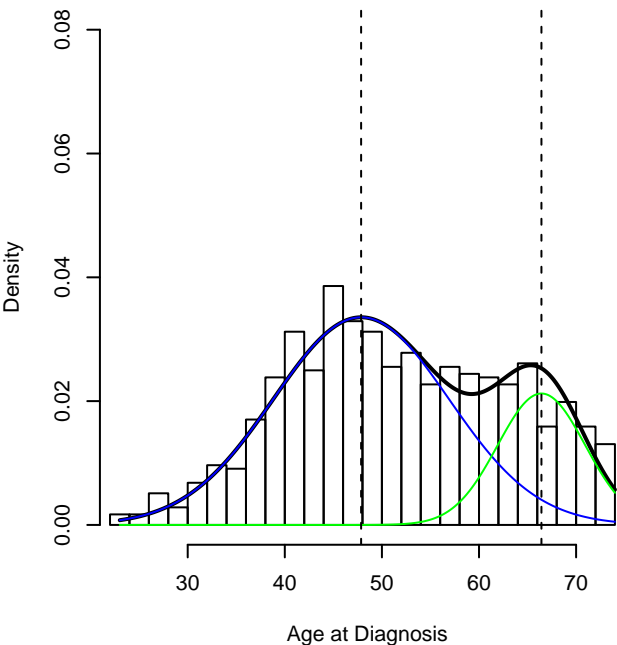

ER <10% (n=568)

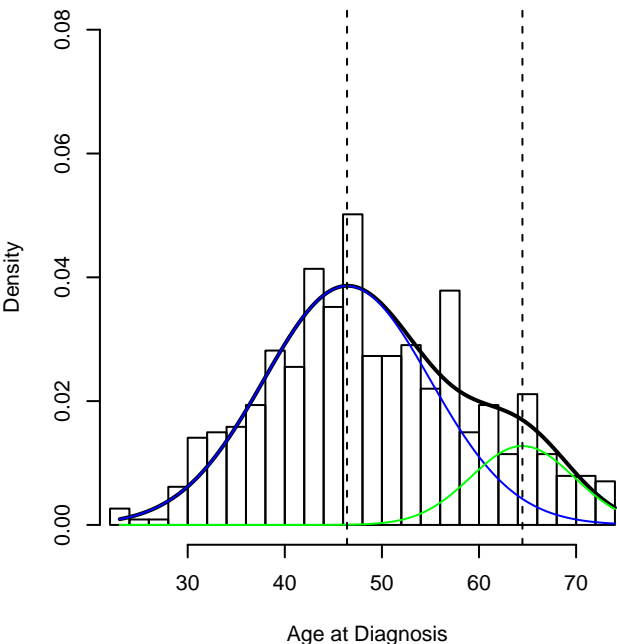

ER >= 10% (n=782)

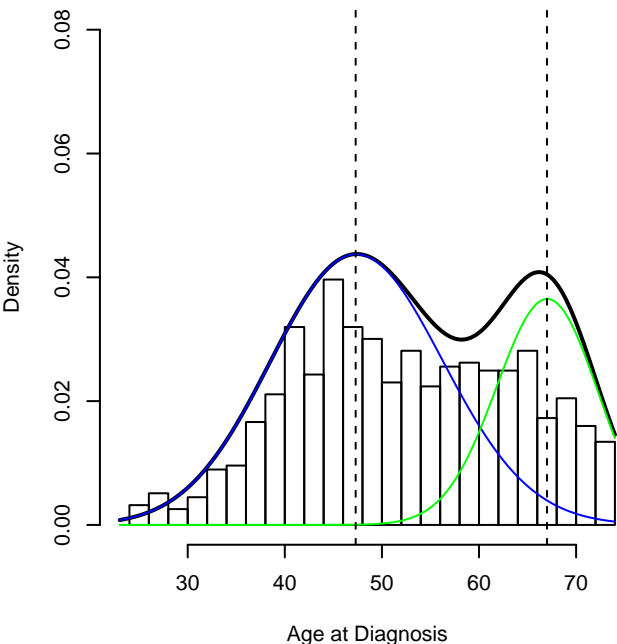

Supplement: Supplementary file 7 — Supplementary Fig. 3: Density plots showing age frequency at diagnosis for invasive breast cancer cases from the Carolina Breast Cancer Study overall and across clinical ER cut points among black women. Supplementary material 7 (PDF 38 kb) [file 10549_2019_5442_MOESM7_ESM.pdf]

**Luminal A (n=3292)**

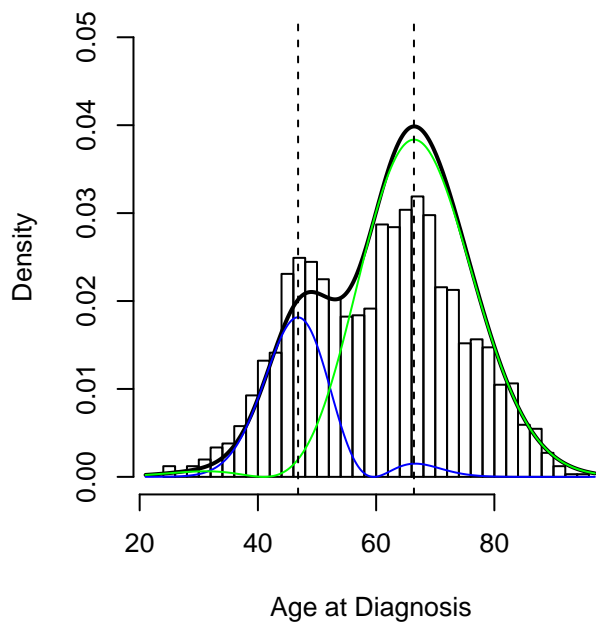

**Luminal B (n=1750)**

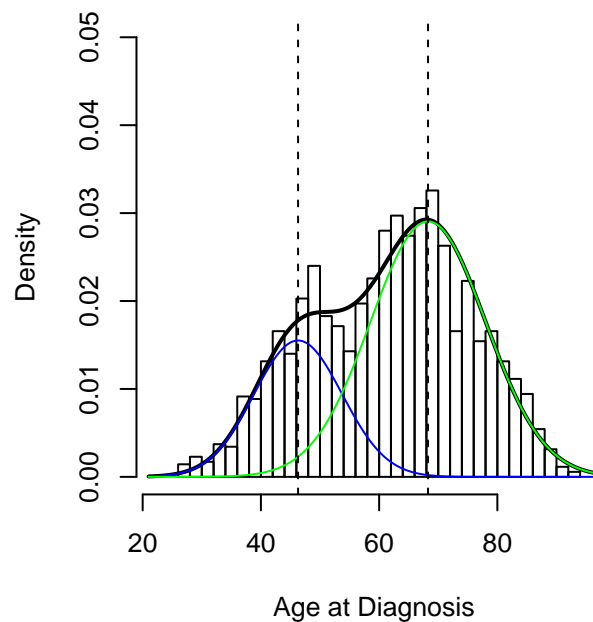

**Her2-enriched (n=917)**

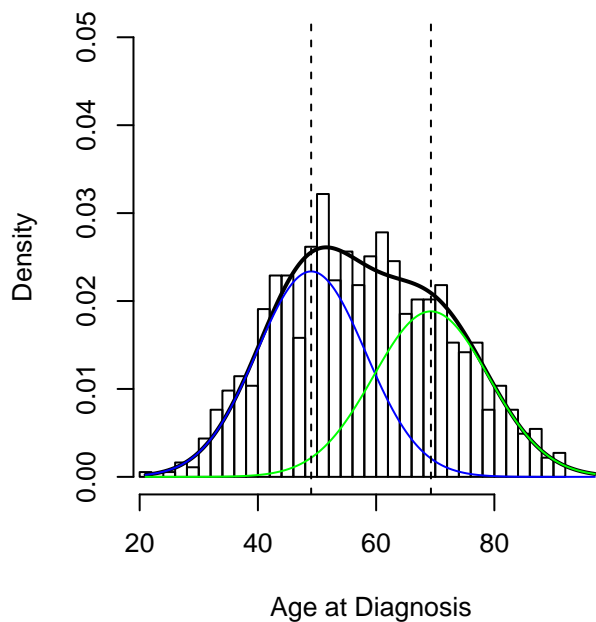

**Basal-like (n=1411)**

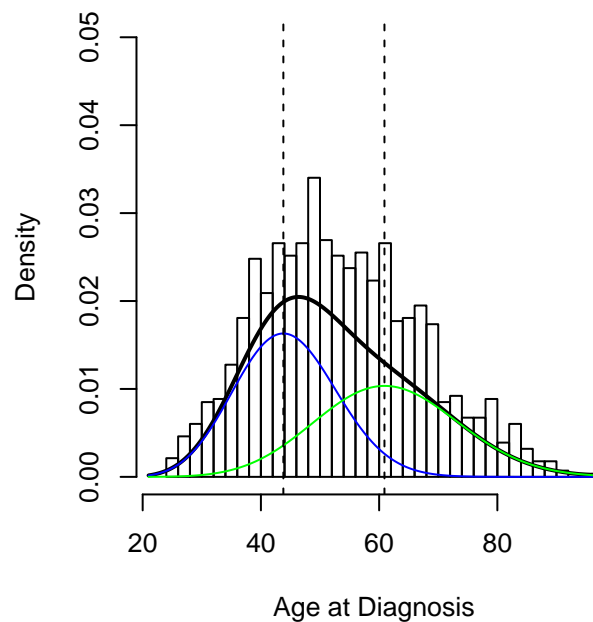

Supplement: Supplementary file 8 — Supplementary Fig. 4: Density plots showing age frequency at diagnosis for invasive breast cancer cases from a pooled publicly-available dataset of 8103 tumors, across PAM50 intrinsic subtype. Supplementary material 8 (PDF 55 kb) [file 10549_2019_5442_MOESM8_ESM.pdf]
