# Supplementary material for: Bimodal age distribution at diagnosis in breast cancer persists across molecular and genomic classifications
Source: Breast Cancer Res Treat. 2019 Sep 18;179(1):185–95. doi: 10.1007/s10549-019-05442-2 (PMC6985047; doi:10.1007/s10549-019-05442-2)
Supplement: Supplementary file 9 — Supplementary Fig. 5: Density plots showing age frequency at diagnosis for invasive breast cancer cases from the Carolina Breast Cancer Study across tumor characteristics. Supplementary material 9 (PDF 67 kb) [file 10549_2019_5442_MOESM9_ESM.pdf]

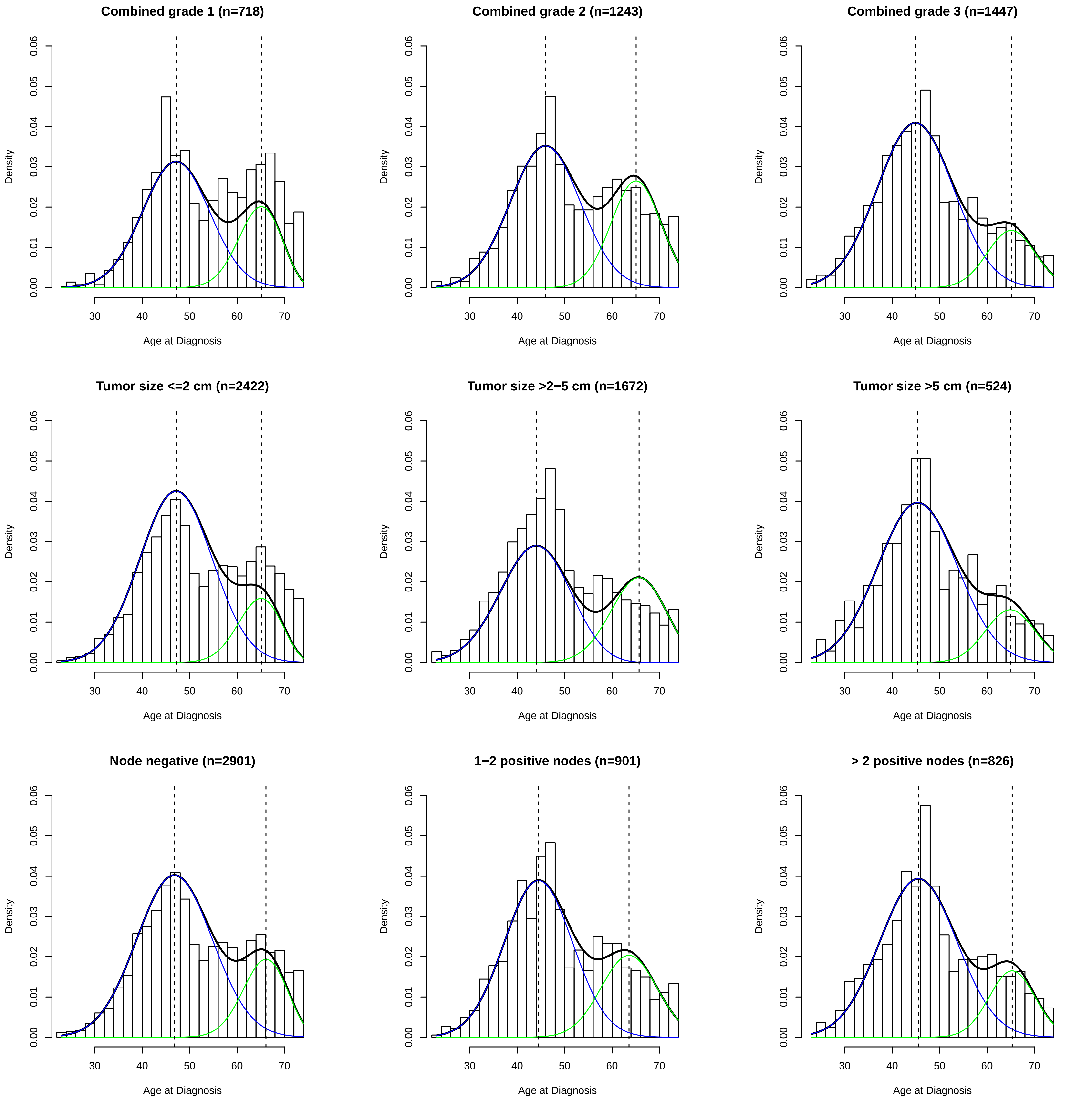
Combined grade 1 (n=718)
Combined grade 2 (n=1243)
Combined grade 3 (n=1447)
Tumor size <=2 cm (n=2422)
Tumor size >2−5 cm (n=1672)
Tumor size >5 cm (n=524)
Node negative (n=2901)
1−2 positive nodes (n=901)
> 2 positive nodes (n=826)
Density
0.00
0.01
0.02
0.03
0.04
0.05
0.06
Age at Diagnosis
30
40
50
60
70
